# A Nanopore-Structured Nitrogen-Doped Biocarbon Electrocatalyst for Oxygen Reduction from Two-Step Carbonization of *Lemna minor* Biomass

**DOI:** 10.1186/s11671-016-1489-3

**Published:** 2016-05-25

**Authors:** Chaozhong Guo, Zhongbin Li, Lidan Niu, Wenli Liao, Lingtao Sun, Bixia Wen, Yunqing Nie, Jing Cheng, Changguo Chen

**Affiliations:** Research Institute for New Materials Technology, Chongqing University of Arts and Sciences, Chongqing, 402160 China; College of Chemistry and Chemical Engineering, Chongqing University, Chongqing, 400044 China; School of Materials and Chemical Engineering, Chongqing University of Arts and Sciences, Chongqing, 402160 China; Chongqing Institute for Food and Drug Control, Chongqing, 401121 China

**Keywords:** Nitrogen-doped carbon, Nanopore, Oxygen reduction, Catalyst, *Lemna minor*

## Abstract

So far, the development of highly active and stable carbon-based electrocatalysts for oxygen reduction reaction (ORR) to replace commercial Pt/C catalyst is a hot topic. In this study, a new nanoporous nitrogen-doped carbon material was facilely designed by two-step pyrolysis of the renewable *Lemna minor* enriched in crude protein under a nitrogen atmosphere. Electrochemical measurements show that the onset potential for ORR on this carbon material is around 0.93 V (versus reversible hydrogen electrode), slightly lower than that on the Pt/C catalyst, but its cycling stability is higher compared to the Pt/C catalyst in an alkaline medium. Besides, the ORR at this catalyst approaches to a four-electron transfer pathway. The obtained ORR performance can be basically attributed to the formation of high contents of pyridinic and graphitic nitrogen atoms inside this catalyst. Thus, this work opens up the path in the ORR catalysis for the design of nitrogen-doped carbon materials utilizing aquatic plants as starting precursors.

## Background

The increasing environmental pollution and depletion of fossil fuels have obliged peoples to develop green and clean energy sources. The fuel cell technology is considered as a very promising energy-conversion device all the time, because it possesses high-efficiency and environment-friendly characteristics [1]. Currently, one of the main factors that hinder the commercialization of fuel cells is sluggish reaction kinetics of cathode oxygen reduction reaction (ORR) [2–4]. Up to date, noble-metal Pt-based materials are unanimously thought as the state-of-the-art ORR catalysts [5]. Unfortunately, high cost and limited natural supply of platinum seriously restrict the development of conventional Pt/C catalysts and the sustainable application of fuel cells [6]. Therefore, exploiting price-moderate and resource-rich catalysts for ORR to substitute for Pt-based catalysts has remained a great challenge.

In the past decades, numerous research studies were largely focused on the synthesis of various non-Pt catalysts such as metallic oxide catalysts [7], non-precious-metal (Me-N_*x*_/C) catalysts [8], heteroatom-doped carbon catalysts [9], and graphene-porphyrin MOF composite [10] in order to replace the precious Pt catalyst. Nitrogen-doped carbon materials with different morphologies, including N-doped graphene [11], N-doped carbon nanotube [12], N-doped carbon sphere [13], and N-doped carbon nanoweb [14, 15] were rapidly developed as effective ORR catalysts in various electrolytes. Besides, 2D sandwich-like zeolitic imidazolate framework (ZIF)-derived graphene-based nitrogen-doped porous carbon sheets (GNPCS ) were obtained by in situ growing ZIF on graphene oxide [16]. Compared to the commercial Pt/C catalyst, the GNPCSs show comparable onset potential, higher current density, and especially, an excellent tolerance to methanol and superior durability in the ORR. Recently, efficient ORR catalysts derived from renewable and earth-abundant plant biomass are highly desirable. A large number of conveniently available biocarbon catalysts for ORR were developed from organic plants, e.g. *Typha orientalis* [17], monkey grass [18], *Ipomoea aquatica* [19], ginkgo leaf [20], soybean [21], and amaranthus waste [22], which can exhibit reasonable ORR catalytic performances. We previously synthesized a novel N-doped carbon nanomaterial with a superior ORR activity from thermal transformation of enoki mushroom biomass at high temperatures [23]. Our results interestingly indicated that the plants rich in biological protein could largely benefit the formation of N-containing active centers for the ORR and help to enhance the electrocatalytic activity in both alkaline and acidic electrolytes.

*Lemna minor* is a species of *Lemna* with a subcosmopolitan distribution, native throughout most of Africa, Asia, Europe. and North America. It is an important food resource for many fish and birds (notably ducks), because it is rich in crude proteins. However, *L. minor* is structurally adapted to grow quickly and sometimes may be considered a pest or organic waste. Therefore, we here report a new strategy to design the nitrogen-doped biocarbon material by two-step carbonization of *L. minor* as a starting material under N_2_ protection. The electrocatalytic activity towards the ORR and long-term stability of this material were evaluated by the rotation disk electrode, and its structural characteristics were also examined by X-ray photoelectron spectroscopy, Raman spectroscopy, and nitrogen-sorption measurements.

## Methods

### Material Synthesis

*L. minor* (common duckweed, CDW) was completely dried at 120 °C in a drying oven, and then ground in an agate mortar for 1 h. 1.0 g of CDW dried powder was first carbonized at 300 °C for 2 h to promote thermal decomposition of bioproteins as far as possible, and the obtained carbonaceous material was marked for CDW-300. Subsequently, the CDW-300 was heat-treated in a tubular furnace at 700 °C for 2 h and then cooled to room temperature. The produced samples were further leached by 1.0 mol l^−1^ HCl solution, which was labeled as NPNC-700. As a control, the same method was applied to synthesize NPNC-600 and NPNC-800. All the heat treatment processes were carried out in nitrogen atmosphere with a heating rate of 10 °C min^−1^.

### Physical Characterization

The surface and morphology were visualized using electron microscopy facility (JEOL FE-2010 high-resolution microscope operated at 200 kV). The Raman spectra were recorded with a Renishaw inVia unit using the Ar ion laser with an excitation wavelength of 514.5 nm. X-ray photoelectron spectroscopy (XPS) analysis was studied using a VG Scientific ESCALAB 220 iXL spectrometer with an Al Kα (hv = 1486.69 eV) X-ray source. Nitrogen adsorption and desorption isotherms were measured at 77 K using Micromeritics ASAP 2010 Analyzer (USA) to obtain the Brunauer-Emmett-Teller (BET) surface area and pore size distribution.

### Electrochemical Measurements

Electrochemical experiments were conducted at 30 °C on a Zennium-E electrochemical workstation (Zahner, Germany) with a three-electrode system. A Pt sheet (1.0 cm^2^ geometric area) and a saturated calomel electrode (SCE) were used as auxiliary and reference electrodes, respectively. A rotation disk electrode (RDE) with a glass carbon (GC) electrode (4 mm diameter, American Model 636 Princeton Applied Research, 0.1256 cm^2^ geometric area) was used as a working electrode. The modified-GC working electrode was prepared by coating it with the catalyst ink. Typically, 5-μl catalyst ink, well dispersed by 0.5 wt% nafion/isopropanol solution, was dropped onto the GC-RDE surface and then dried in air. About 50 μg of the carbon catalyst, except 20 wt% Pt/C-ETK catalyst (25 μg), was loaded on the GC-RDE surface. All of the electrode potentials in this study are quoted versus a reversible hydrogen electrode (RHE). All RDE experiments were performed at a scan rate of 5 mV s^−1^ in O_2_-saturated 0.1 mol l^−1^ KOH solution under different rotation rates. The Koutecky-Levich (K-L) equation was used to calculate the number of electron transfer [24].

$$ \frac{1}{j_{\mathrm{d}}}=\frac{1}{j_{\mathrm{k}}}+\frac{1}{0.62\mathrm{n}F{C}_{\mathrm{O}}{D}_{\mathrm{O}}^{2/3}{\nu}^{-1/6}{\omega}^{1/2}} $$

where *j*_d_ and *j*_k_ are the measured current density and kinetic limiting current density, respectively, *F* is the Faradaic constant (C mol^−1^), *C*_O_ is the O_2_ saturation concentration in the aqueous solution (mol cm^−3^), *D*_O_ is the O_2_ diffusion coefficient in the aqueous solution (cm^2^ s^−1^), *v* is the kinetic viscosity of the solution (cm^2^ s^−1^), *ω* is the electrode rotation rate (rpm), and 0.62 is a constant when the rotation rate is expressed in rpm.

## Results and Discussion

### Structural and Morphology Characterizations

**Fig. 1:**
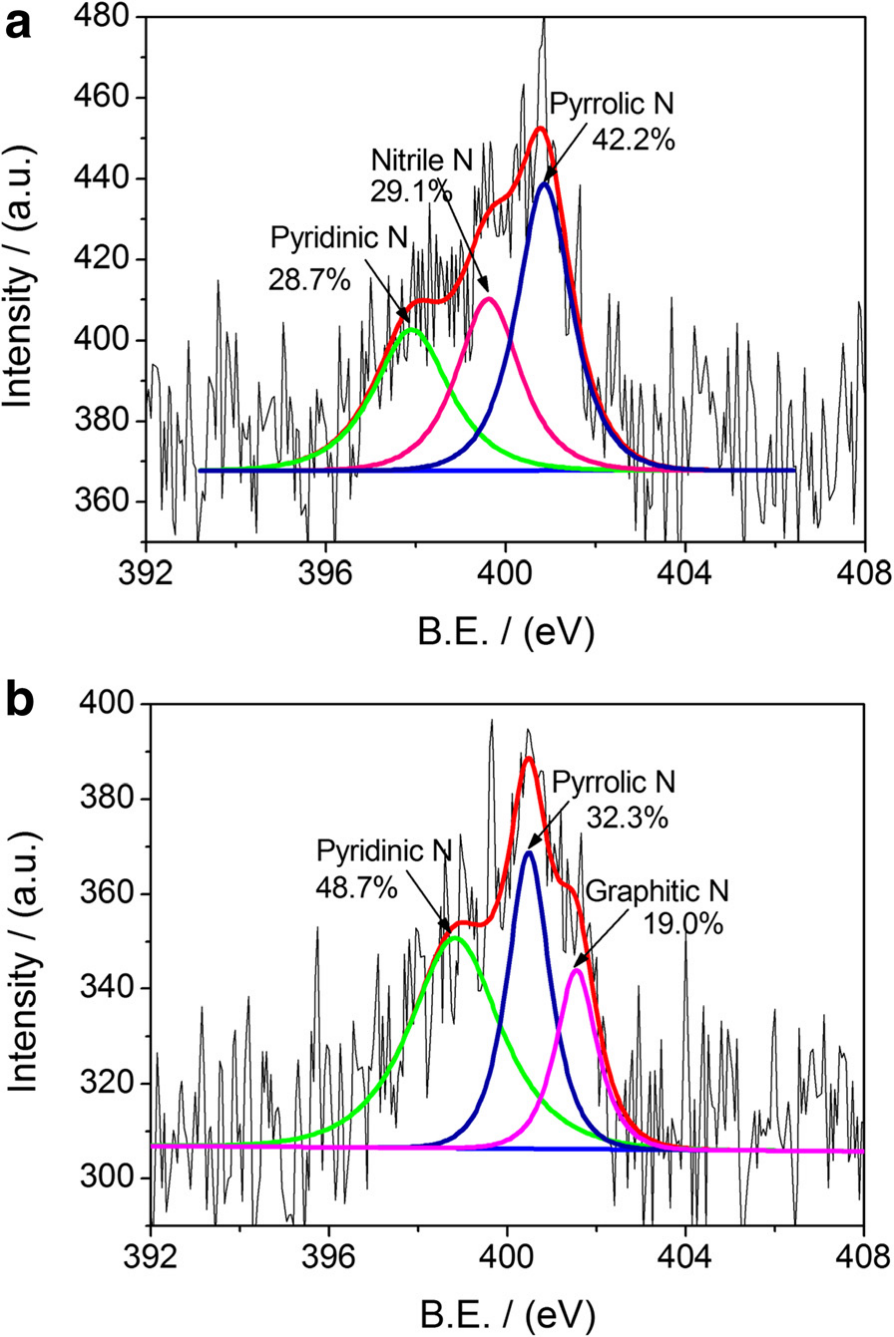
**a** High-resolution N1s XP narrow-scan spectrum of CDW-300; **b** High-resolution N1s XP narrow-scan spectrum of NPNC-700

**Fig. 2:**
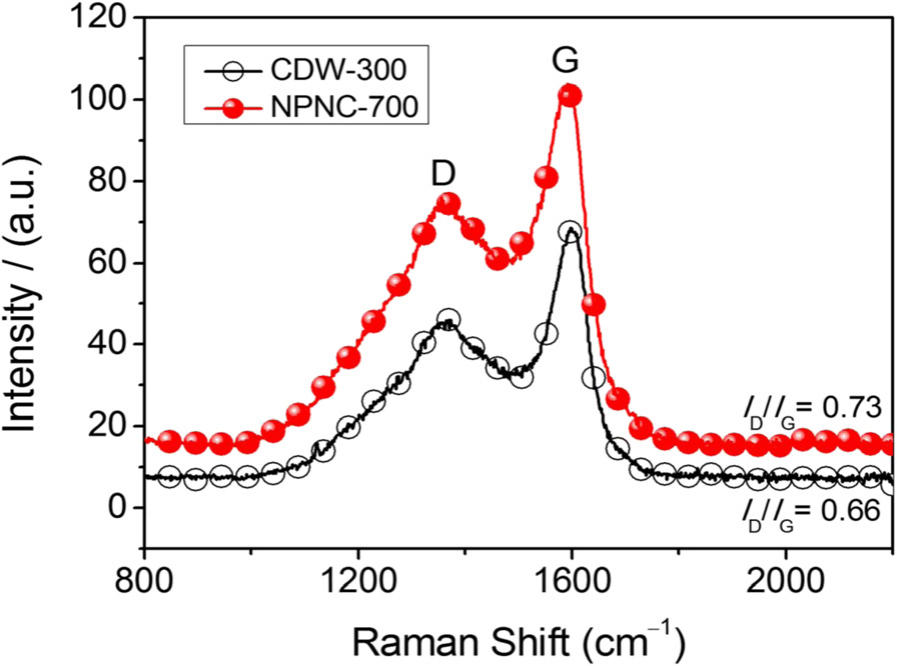
Raman spectra of CDW-300 and NPNC-700

**Fig. 3:**
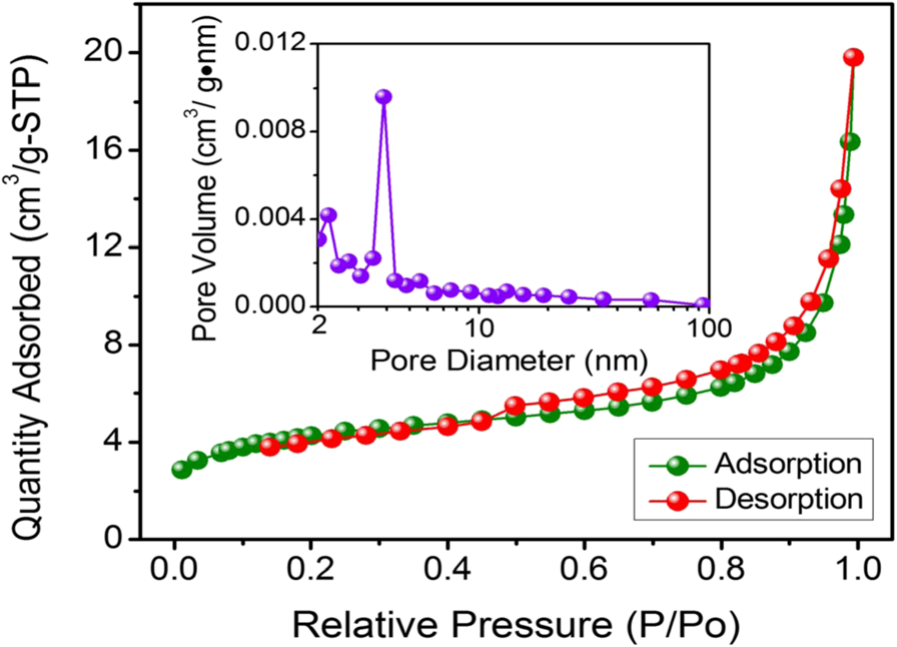
N_2_-adsorption/desorption isotherm and corresponding pore size distribution of NPNC-700

**Fig. 4:**
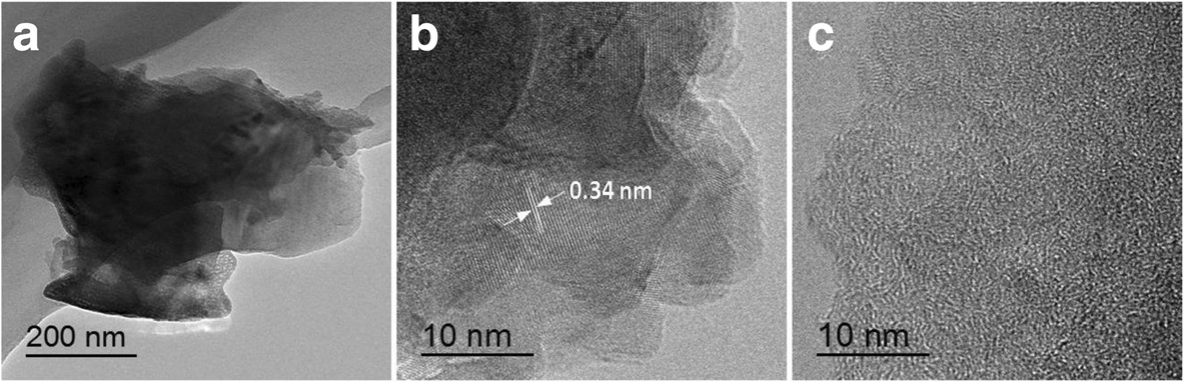
**a**–**c** High-resolution transmission electron microscopy (HRTEM) images of NPNC-700

We firstly probed the chemical status of nitrogen atoms in the NPNC-700 catalyst by the analysis of N1s narrow-scan XP spectrum, as shown in Fig. 1a. The XP N1s spectrum of CDW-300 was also examined for comparison in Fig. 1b. The total nitrogen content in atomic ratio, which was determined via elemental analysis, was 2.80 and 2.15 % for CDW-300 and NPNC-700, respectively, suggesting that the pyrolysis process at high temperature clearly led to the loss of nitrogen inside carbon materials. The characteristic peak of the nitrogen atom indicates the disruption of π-conjugation on the outer layers of the carbon lattice. The XP N1s spectrum of the CDW-300 precursor can be deconvoluted into three peaks, which are attributed to pyridinic, nitrile, and pyrrolic nitrogen atoms, respectively, in the carbon lattice [23]. However, the nitrile nitrogen atom is not observed at the XP N1s spectrum of the NPNC-700 catalyst. In contrast, a certain ratio (19.0 at.%) of the graphitic nitrogen atom is produced, which obviously displays that the heat-treatment process at 700 °C causes the rapid decomposition of the nitrile and pyrrolic nitrogen atoms owing to their instability [25]. The atomic ratio of the pyridinic nitrogen atom is largely increased from 28.7 to 48.7 %, which may play a critical role in the enhancement of the electrocatalytic activity [26]. The Raman spectra of CDW-300 and NPNC-700 were used to investigate the defect sites and disordered structures. In Fig. 2, each Raman spectrum was deconvoluted into two components, which exhibited the characteristic “D” and “G” peaks, respectively. The D band (Lorentzian, located at ~1350 cm^−1^) is disorder-induced, which corresponds to the sp^3^ defect sites on the graphitic plane, including vacancy and heteroatoms. The G band (Lorentzian, located at ~1580 cm^−1^) is commonly observed for all graphitic structures and attributed to the E2g vibration mode present in the sp^2^-bonded graphitic carbons [27]. No significant shifts in the position of the “D” and “G” bonds as a function of the temperature were observed. The intensity ratio of the D peak to the G peak, namely the *I*_D_/*I*_G_ ratio, can provide the indication of the amount of structural defects and a quantitative measure of edge plane exposure. It is importantly found that the *I*_D_/*I*_G_ ratio (0.73) of NPNC-700 is slightly larger than that of CDW-300 (0.66), which indicates that more nitrogen heteroatoms are doped into the graphene layers in the graphite structure of NPNC-700, producing more structural defects by nitrogen-doping [11, 28]. We further measured the N_2_-sorption isotherms of the NPNC-700 catalyst (Fig. 3). It exhibits a type II isotherm curve, which usually comes from nonporous particles [29]. The Brunauer-Emmet-Teller (BET) specific surface area was determined to be only 15.1 m^2^ g^−1^. The total pore volume is 0.019 cm^3^ g^−1^, and the surface of NPNC-700 predominantly consists of nanopores with an average size of ~5.0 nm (inset of Fig. 3). Numerous nanopores can be clearly observed at the TEM image of NPNC-900 (Fig. 4a,). The interplanar spacing in graphite lattice of the NPNC-700 catalyst can be calculated to about 0.34 nm (Fig. 4b). The HRTEM image (see Fig. 4c) about the edged plane of NPNC-700 displays the disordered graphite structure. Besides, some structural defects can be expectedly found on the inner plane of NPNC-700, which may be attributed to the doping of nitrogen atoms into the carbon structure [23].

### Electrocatalytic Activity for Oxygen Reduction

**Fig. 5:**
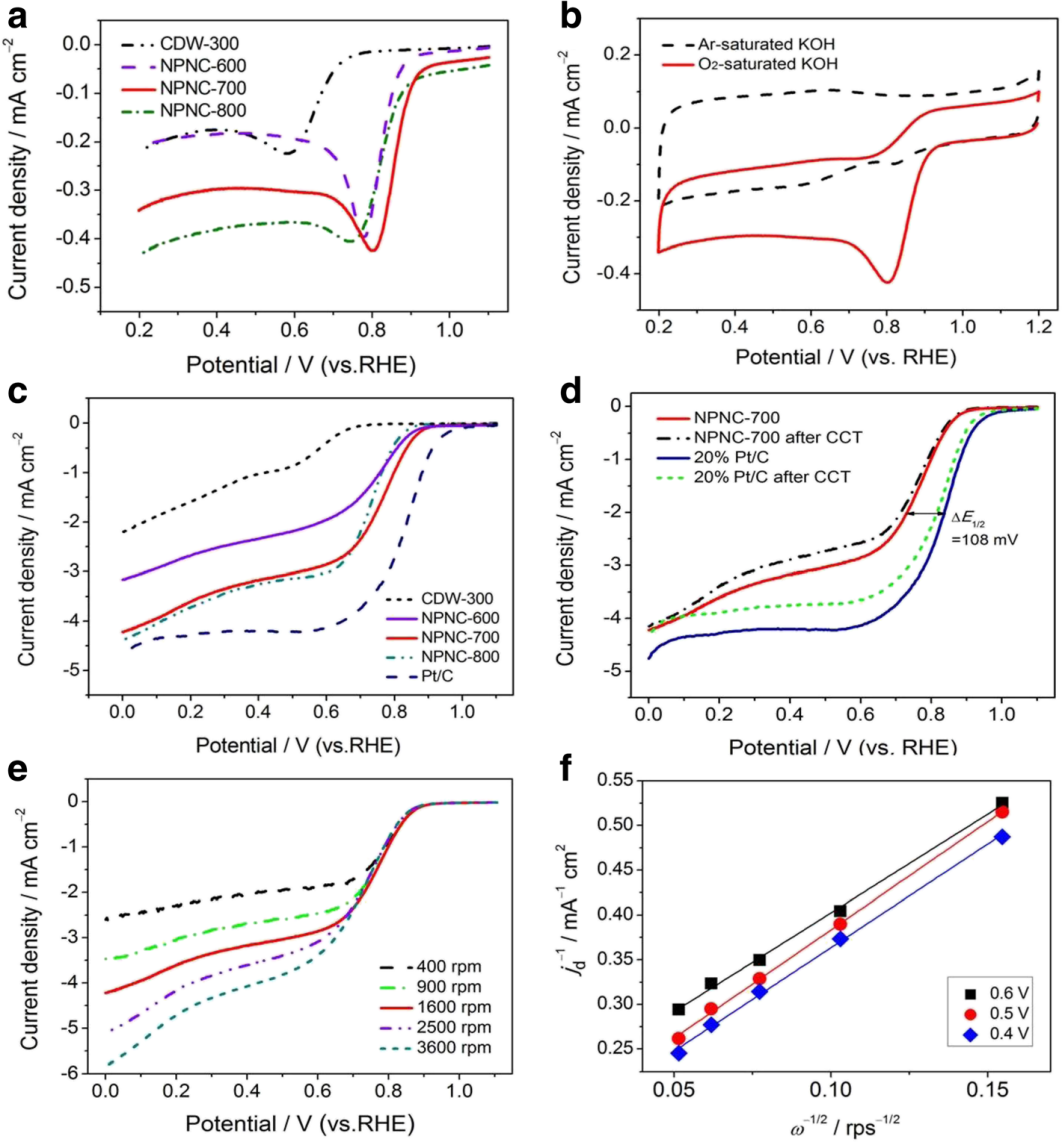
**a** LSV curves for ORR of CDW-300, NPNC-600, NPNC-700, and NPNC-800 in O_2_-saturated 0.1 mol l^−1^ KOH electrolyte. **b** CV curves for NPNC-700 in Ar versus O_2_ saturated 0.1 mol l^−1^ KOH electrolyte. **c** ORR polarization curves of CDW-300, NPNC-600, NPNC-700, NPNC-800, and 20 wt% Pt/C in 0.1 mol l^−1^ KOH electrolyte under O_2_ protection. **d** ORR polarization curves of NPNC-700 and 20 wt% Pt/C before and after the CCT. **e** ORR polarization curves of NPNC-700 in O_2_-saturated 0.1 mol l^−1^ KOH electrolyte at different rotation rates. **f** Koutecky-Levich plots of *j*_d_^−1^ versus *ω* ^−1/2^ obtained from **e**

Figure 5a shows linear sweep voltammetry (LSV) curves of different carbon materials in O_2_-saturated 0.1 mol l^−1^ KOH solution. Compared with the LSV curve of CDW-300 precursor, it can be found that the second pyrolysis process can obviously enhance the ORR catalytic activity of the final product. The NPNC-700 has exhibited the best electrocatalytic activity with an ORR peak potential of about 0.80 V (versus RHE); however, others display relatively poor catalytic activity. We further assessed the activity of the NPNC-700-catalyzed electrode in Ar versus O_2_-saturated electrolyte by cyclic voltammetry (CV), as indicated in Fig. 5b. Expectedly, the CV curve of NPNC-700 in an aqueous solution of 0.1 mol l^−1^ KOH under Ar protection has displayed a virtually featureless curve. This result can qualitatively explain the ORR activity of the NPNC-700 catalyst in an alkaline electrolyte. In order to further study the ORR catalytic performance of prepared carbon materials, the RDE was applied to examine the electrocatalytic activity and long-term stability. In Fig. 5c, we also find that the NPNC-700 catalyst relatively demonstrates a better catalytic activity in terms of onset potential (*E*_ORR_), half-wave potential (*E*_1/2_), and limited current density (*j*_d_), compared with other carbon-based catalysts in this work. The onset and half-wave potentials for the ORR of the NPNC-700 catalyst are around 0.93 and 0.74 V, slightly lower than those of the 20 wt% Pt/C catalyst and several reported carbon-based catalysts [13–15, 21–23]. We interestingly found that the mass-transfer limited current density of NPNC-800 is slightly higher in the LSV measurement. It may be attributed to a higher content of the graphitic-N species in the NPNC-800 catalyst. Lai et al [30] previously proposed that the ORR activity of the catalyst was found to be dependent on the graphitic N content which determined the limiting current density. Remarkably, no noticeable changes in the onset potential and limited current density of the ORR were observed on the NPNC-700-catalyzed electrode, and the ORR half-wave potential on this electrode was negatively shifted by only 18 mV, after 1000 continuous cycling tests (CCT) in the O_2_-satured alkaline electrolyte, suggesting the excellent long-term stability of the NPNC-700 catalyst, as shown in Fig. 5d. However, the ORR electrocatalytic activity of the Pt/C catalyst was largely reduced after the CCT. The ORR half-wave potential on the Pt/C-catalyzed electrode was negatively shifted by about 30 mV (Fig. 5d), indicating that the relatively poor stability of the Pt/C comparing with the prepared NPNC-700 catalyst.

To explain the ORR catalytic mechanism of the NPNC-700 catalyst in the alkaline electrolyte, we also measured the ORR polarization curves in 0.1 mol l^−1^ KOH at different rotation speeds (400–3600 rpm), as displayed in Fig. 5e. The ORR current densities measured on NPNC-700 increase with the increase of RDE rotation rates. The good linearity of Koutecky-Levich (K-L) plots (Fig. 5f) and near parallelism of fitting lines synergistically show the first-order dependence of the ORR kinetics and similar electron transfer numbers for ORR at different potentials. The average electron transfer number (*n*) was calculated to be ~3.9 for NPNC-700 and the average kinetic current density (*j*_k_) was calculated to be ~6.7 mA cm^−2^ for NPNC-700, respectively, based on the slopes and intercepts of K-L plots obtained at 0.5–0.7 V versus RHE. Hence, the ORR on NPNC-700 proceeds mainly with a four-electron reduction pathway, very similar to the ORR electro-catalyzed by the Pt/C catalyst [29]. Results show that the NPNC-700 catalyst is a promising candidate for commercial Pt/C catalysts in the alkaline electrolytes.

In recent years, the exploration of active sites for the ORR in nitrogen-containing carbon-based catalysts was a hot topic all the time. Numerous studies were mainly focused on the geometry of the surface nitrogen-containing groups and their significant correlations with the electrocatalytic activity [13, 26, 27]. However, up to date, it is only confirmed that the doping of nitrogen atoms into the graphite lattice can promote the electrocatalytic activity thanks to the formation of chemically active and localized areas of higher electron density [31]. In this study, we interestingly find that a high content of pyrrolic nitrogen in the catalyst has not played a key role in the ORR performance, but the formation of pyridinic and graphitic nitrogen during high-temperature pyrolysis can synergistically enhance the ORR electrocatalytic performance. Lai and his co-workers previously proposed that the activity of the doped carbon catalysts was found to be dependent on the graphitic nitrogen content, while the pyridinic nitrogen content improved the onset potential for ORR [30]. In RDE experiments, the limiting current density of NPNC-700 was much higher than that of CDW-300 and the onset potential for ORR of NPNC-700 was more positive than that of CDW-300. These results may exactly correspond to the XPS analyses for N1s region of CDW-300 and NPNC-700. Thus, it can be reasonably concluded that the pyridinic and graphitic nitrogen atoms may be the catalytically active centers for the ORR in our catalysts.

## Conclusions

In summary, a novel nitrogen-containing biocarbon material with nanoporous structures was synthesized by two-step carbonization of *L. minor* under the nitrogen atmosphere. This material exhibits the ORR electrocatalytic activity with an onset potential of around 0.93 V, and the excellent cycling stability in alkaline medium. The electron transfer number of the ORR on our catalyst was calculated to be around 3.9, almost identical to that on the commercial Pt/C catalyst. The pyridinic and graphitic nitrogen can be largely responsible for the enhancement of the ORR activity, which may be the catalytically active centers for the ORR. Our results provide a new idea for the design of renewable biocarbon materials from water plants, helping to construct ORR-active electrocatalysts.
